# Clinical spectrum, treatment and outcome of children with suspected diagnosis of chronic inflammatory demyelinating polyradiculoneuropathy

**DOI:** 10.1016/j.nmd.2018.06.001

**Published:** 2018-09

**Authors:** A. Silwal, M. Pitt, R. Phadke, K. Mankad, J.E. Davison, A. Rossor, C. DeVile, M.M. Reilly, A.Y. Manzur, F. Muntoni, P. Munot

**Affiliations:** a The Dubowitz Neuromuscular Centre, UCL Great Ormond Street Institute of Child Health, 30 Guilford St, London, and MRC Centre for Neuromuscular Diseases & Neuroscience Unit, Great Ormond Street Hospital, London, UK; b Neuroradiology Department, Great Ormond Street Hospital for Children NHS Foundation Trust, London, UK; c Metabolic Medicine, Great Ormond Street Hospital for Children NHS Foundation Trust, London, UK; d MRC Centre for Neuromuscular Diseases, UCL Institute of Neurology and National Hospital for Neurology and Neurosurgery, Queen Square, London, UK

**Keywords:** Inflammatory neuropathy, CIDP, Immuno-modulatory therapy

## Abstract

Chronic inflammatory demyelinating polyradiculoneuropathy (CIDP) is a treatable chronic disorder of the peripheral nervous system. We retrospectively studied 30 children with a suspected diagnosis of CIDP. The diagnosis of CIDP was compared against the childhood CIDP revised diagnostic criteria 2000. Of the 30 children, five did not meet the criteria and four others met the criteria but subsequently had alternative diagnosis, leaving a total of 21 children (12 male) with CIDP as the final diagnosis. Thirteen children presented with chronic symptom-onset (>8 weeks). The majority presented with gait difficulties or pain in legs (*n* = 16). 12 children (57%) met the neurophysiological criteria and 18/19 (94%) met the cerebrospinal fluid criteria. Nerve biopsy was suggestive in 3/9 (33%), with magnetic resonance imaging supportive in 9/20 (45%). Twenty-one children received immuno-modulatory treatment at first presentation, of which majority (*n* = 19, 90%) received IVIG (immunoglobulin) monotherapy with 13 (68%) showing a good response. 8 children received second line treatment with either IVIG or steroids or plasmapharesis (PE) and 4 needed other immune-modulatory agents. During a median follow-up of 3.6 years, 9 (43%) had a monophasic course and 12 (57%) had a relapsing–remitting course. At last paediatric follow up 7 (33%) were off all treatment, 9 (43%) left with no or minimal residual disability and 6 (28%) children were walking with assistance (*n* = 3) or were non-ambulant (*n* = 3). Our review highlights challenges in the diagnosis and management of paediatric CIDP. It also confirms that certain metabolic disorders may mimic CIDP.

## Highlights

• The diagnosis of CIDP can be challenging.
• In our cohort 52% were diagnosed as CIDP on re-evaluation.
• Cranial nerve abnormality is rare and may be only presenting symptom.
• Children require long-term follow up as the course may be protracted.
• With early treatment majority have good recovery and maintain ambulation.

## 1 Introduction

Chronic inflammatory demyelinating polyradiculoneuropathy (CIDP) is an immune mediated treatable disorder of the peripheral nerves, with predominant motor involvement and an insidious onset over months, or recurrent episodes [1]. Children present with slowly progressive or relapsing episodes of gait difficulty, distal symmetric weakness and sometimes paraesthesiae [2]. Reflexes are absent or depressed. Laboratory findings include elevated cerebrospinal fluid (CSF) protein with no increase in cells. Electrophysiological and pathological studies show evidence of demyelination. The diagnosis of CIDP is usually straightforward, but atypical presentations can represent a significant diagnostic challenge [3]. Differential diagnoses include its acute counterpart, Guillain–Barré syndrome (GBS), as well as hereditary and metabolic causes of childhood polyneuropathy [2].

CIDP results in segmental and multifocal demyelination that may induce axonal loss with time. Goals of diagnosis and management are to control the inflammation and therefore prevent axonal loss and the resulting disability. Although in some the course is monophasic with complete recovery, in others it can be slowly-progressive, or relapsing-remitting resulting in prolonged morbidity and sometimes permanent disability [1]. However, if diagnosed and treated promptly, children with CIDP typically respond well to immuno-modulatory therapy and the prognosis for remission of neurologic deficits may be good.

CIDP is less common in children than adults and hence knowledge of the clinical characteristics, response to treatment, and prognosis are often based on several small paediatric series. In this series we attempt to describe the presentation, diagnosis, response to treatment and long-term outcome of childhood CIDP.

## 2 Methods

Children seen in the neuromuscular service at a single tertiary unit from 1992 to 2015 with a suspected diagnosis of CIDP were identified from our neuromuscular and neurophysiology databases and their medical records were reviewed.

Revised diagnostic criteria for childhood CIDP proposed by Nevo et.al. [1] (Table 1) were used to define criteria for diagnosing children with CIDP. This was based on mandatory clinic criteria which includes two features (i) Progression of muscle weakness in proximal and distal muscles of upper and lower extremities over at least 4 weeks, or alternatively when rapid progression (GBS like presentation) is followed by relapsing or protracted course (more than 1 year), and (ii) Areflexia or hyporeflexia. Apart from the requirement of mandatory clinical criteria; for confirmed CIDP diagnosis specific electrodiagnostic (EDT) and CSF features were also required, whereas 1 of 3 paraclinical findings (EDT, CSF or biopsy features) was required for possible CIDP diagnosis.

**Table 1:** Revised diagnostic criteria for childhood (2000)–CIDP.

**1. Confirmed CIDP**
(i) Mandatory clinical features.
(ii) Electrodiagnostic and CSF features.
**2. Possible CIDP**
(i) Mandatory clinical features.
(ii) One of the 3 laboratory findings
**Exclusion criteria**
1. Clinical features or history of a hereditary neuropathy, other diseases or exposure to drugs or toxins that are known to cause peripheral neuropathy.
2. Laboratory findings (including nerve biopsy or DNA studies) that show evidence for a different etiology other than CIDP.
3. Electrodiagnostic features of abnormal neuromuscular transmission, myopathy or anterior horn cell disease
**- Mandatory clinical criteria:**
• Progression of muscle weakness in proximal and distal muscles of upper and lower extremities over at least 4 weeks, or alternatively when rapid progression (GBS like presentation) is followed by relapsing or protracted course (more than 1 year).
• Areflexia or hyporeflexia.
**- A.1. Major laboratory features: Electrophysiological criteria/CSF studies/N.biopsy**
**A.1.1. Electrophysiological criteria**
Must demonstrate at least three of the following four major abnormalities in motor nerves (or 2 of the major plus 2 of the supportive criteria):
A.1.1.1. Major
1. Conduction block or abnormal temporal dispersion in one or more motor nerves at sites not prone to compression.
a. Conduction block: at least 50% drop in negative peak area or peak-to-peak amplitude of proximal compound muscle action potential (CMAP) if duration of negative peak of proximal CMAP is <130% of distal CMAP duration.
b. Temporal dispersion: abnormal if duration of negative peak of proximal CMAP is >130% of distal CMAP duration.
Recommendations: (a) Conduction block and temporal dispersion can be assessed only in nerves where amplitude of distal CMAP is >1 mV. (b) Supramaximal stimulation should always be used.
2. Reduction in conduction velocity (CV) in two or more nerves: <75% of the mean minus 2 standard deviations (SD) CV value for age.
3. Prolonged distal latency (DL) in two or more nerves:> 130% of the mean 12 SD DL value for age.
4. Absent F waves or prolonged F wave minimal latency (ML) in two or more nerves: >130% of the mean 1 2SD F wave ML for age.
5.Recommendation: F wave study should include a minimum of 10 trials.
** **A.1.1.2. Supportive
** **1. When conduction block is absent, the following abnormal electrophysiological parameters are indicative of non-uniform slowing and thus of an acquired neuropathy:
** 2.** Abnormal median sensory nerve action potential (SNAP) while the sural nerve SNAP is normal.
** **3. Abnormal minimal latency index (TLI) [1].
** 4.** Difference of >10 m/s in motor CVs between nerves of upper or lower limbs (either different nerves from the same limb for example left median versus left ulnar or the same nerve from different sides for example left versus right ulnar nerves).
**A.1.2. Cerebrospinal fluid (CSF studies)**
** •** CSF protein>35 mg/dl.
** •** Cell count<10 cells/mm3.
**A.1.3. Nerve biopsy features**
Nerve biopsy with predominant features of demyelination.

Published reference values for normal paediatric sensory and motor nerve conduction values were used [4]. Evidence for nerve root thickening and/or enhancement on magnetic resonance imaging (MRI) of the lumbosacral spine was not included as a part of the diagnostic criteria (as per 2000 criteria).

Information was obtained on age of onset, time to maximum disability, symptoms at presentation; disease course, immuno-modulatory treatment used and disease activity at last follow up. An attempt was made to identify atypical features in the suspected CIDP cases that later had a diagnostic revision. Clinical deficit was quantified using the Modified Rankin scale (MRS; Table 2) [2] at peak motor disability and last follow up. Disease outcome at last follow up visit was assessed using the CIDP Disease Activity Status (CDAS) scale [5]. Disease relapse was defined as clinical deterioration not associated with weaning immunosuppressant medication and/or wearing-off effects of IVIG or plasma exchange therapy and clinical response to treatment was sub-classed as good, partial and no response as defined by McMillan et.al. [2].

**Table 2:** Modified Rankin Score.

**1**	mild symptoms that do not interfere with any work, school or extracurricular activity
**2**	slight disability (i.e. child has given up one or more activities) but is able to perform all age-appropriate personal care (i.e. dressing, eating) and complex tasks (i.e. handwriting, age-appropriate food preparation);
**3**	moderate symptoms (i.e. child is still able to walk independently (may require cane or walker) but requires assistance for age-appropriate tasks (see above))
**4**	moderate-to-severe symptoms (i.e. child is unable to walk (carried by parent and/or wheelchair required) and unable to perform age appropriate personal care)
**5**	severe disability (i.e. patient is bed-ridden and requires constant nursing care), may require intubation and mechanical ventilation
**6**	Death

## 3 Results

**Fig. 1:**
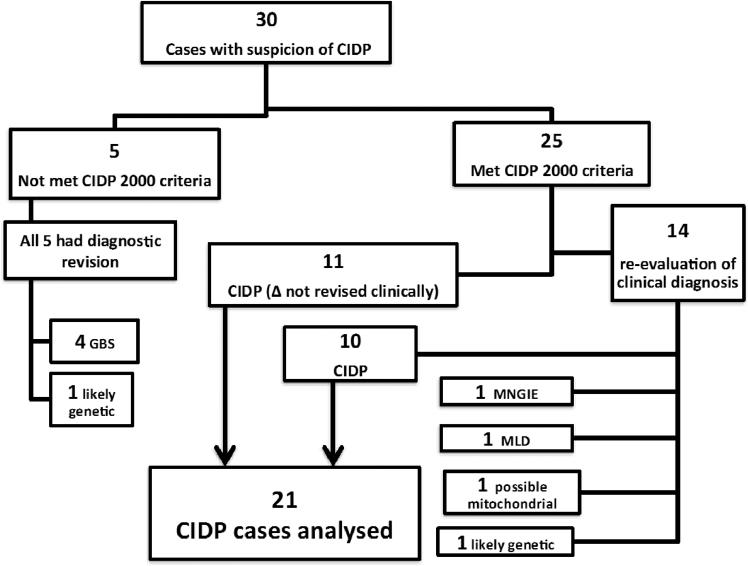
Comparison of CIDP cases with revised CIDP 2000 criteria. GBS: Guillain–Barré syndrome, MNGIE: Mitochondrial Neurogastrointestinal Encephalopathy, MLD: Metachromatic Leukodystrophy

Thirty children with CIDP suspicion were identified (Fig. 1) and their characteristics were compared with the revised diagnostic criteria for childhood CIDP 2000 proposed by Nevo et al. [1]. All but five cases (*n* = 25) met the criteria; ten met the confirmed CIDP criteria and 15 met the possible CIDP criteria. Five cases that did not meet the criteria had a later diagnostic revision to GBS in four cases and a likely genetic cause in the other. Amongst twenty-five that met the criteria four subsequently had alternative diagnosis and were excluded. A total of 21 cases were established to have CIDP and these are detailed as below

### 3.1 Clinical presentation (Supplementary table S1)

The mean age of onset was 7 years and 3 months; range 2–16 years. Symptom onset was acute (<4 weeks) in five, three had a sub-acute (4–8 weeks) and more than 50% had a chronic (>8 weeks) onset (*n* = 13). Two children included in the chronic group had leg pains and foot deformity for more than a year and then became non-ambulant acutely. The interval between symptom onset and maximum disability ranged from <1–44 months. Preceding infectious illness was present in less than half (*n* = 11).

The majority of children presented with gait difficulties (*n* = 11); all subsequently developed 4 limb involvement. All children were areflexic with one hyporeflexic at presentation. 3/21 (14%) had bulbar weakness with weak cry or swallowing/speech difficulty with two others having cranial nerve involvement with anisocoria/optic neuritis and bilateral lower facial weakness, respectively.

### 3.2 Investigations (Supplementary table S2)

Electrophysiology studies done in all the twenty-one children who met the CIDP diagnostic criteria were reviewed; of whom 12 met the electrodiagnostic criteria for CIDP. Parameters that were looked into included presence or absence of conduction block, sensory and motor nerve conduction velocities, distal CMAP latency and F wave latency. A minimum of four to five nerve were studied. In these 12 children that met the electrodiagnostic criteria for CIDP the study was demyelinating in majority (*n* = 8), and both demyelinating and axonal changes in four cases.

Rest of the 9 cases that met the CIDP criteria (confirmed or possible) did not meet all the required major or supportive criteria to meet the electrodiagnostic criteria for CIDP; of these two had only axonal changes.

**Fig. 2:**
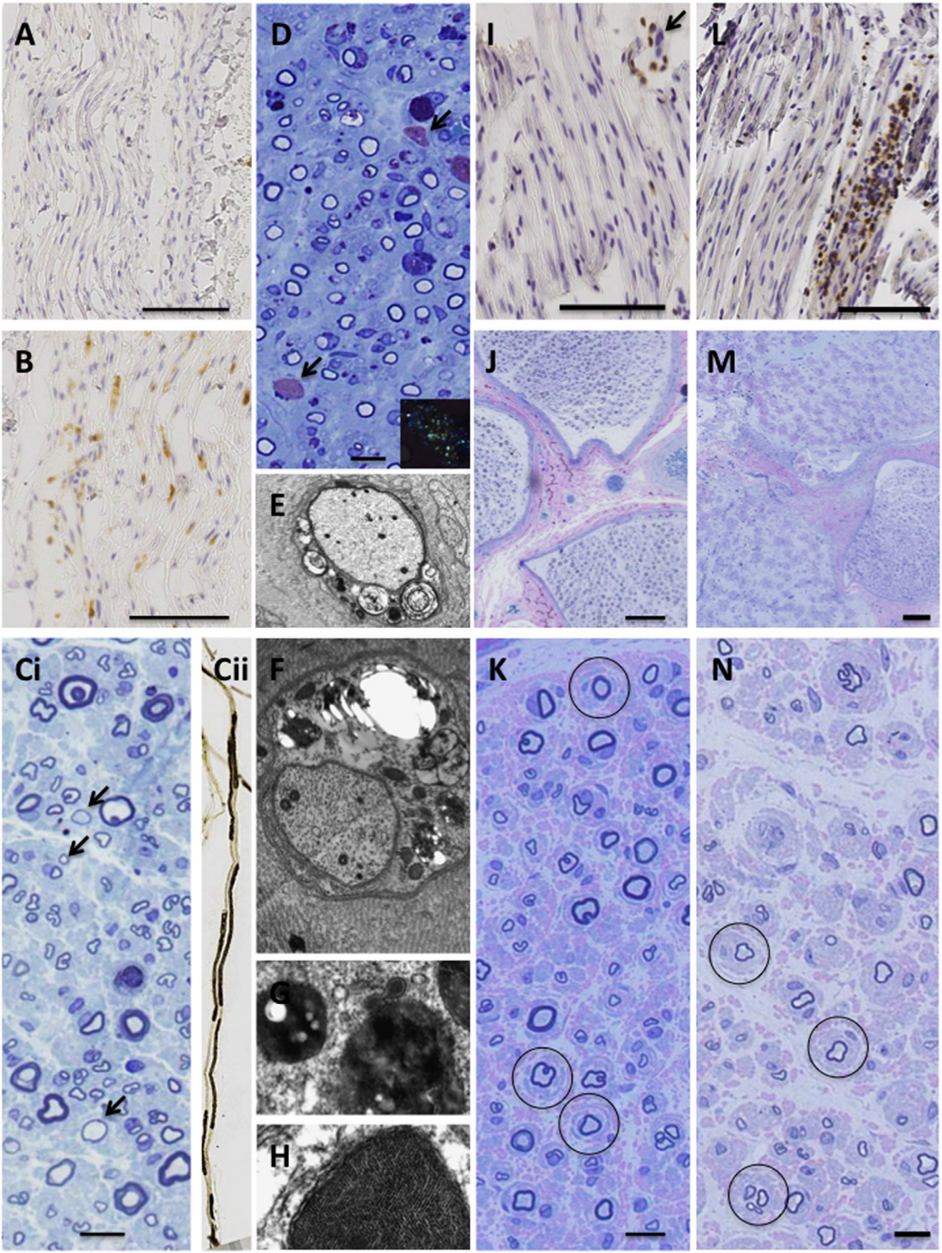
Nerve biopsy image legend: Pathology from peripheral nerve biopsies in P3 at 3 years, 10 months (A-Cii), P7 at 2 years, 6 months (D-G), P27 at 11 years, 4 months (I-K) and P1 at 14 years (L-N). P3: Immunostaining for CD3 (A) shows virtually no inflammatory cells, whilst CD68 (B) highlights mild endoneurial microglial activation, and labeling of occasional myelin ovoids indicating acute axonal degeneration. In a representative fascicle from a resin semi-thin section stained with Toluidine blue (Ci), there are thinly myelinated large and small diameter axons (arrows) admixed with normally myelinated axons. Note the absence of well-formed onion bulbs and regeneration clusters. A teased fibre preparation (Cii) shows a myelinated fibre undergoing paranodal/segmental demyelination. P7: A representative fascicle from a resin semi-thin section stained with Toluidine blue (D). There is uniform hypomyelination affecting majority of axons that show inappropriately thin myelin sheaths. Onion bulbs are not prominent. There is accumulation of metachromatic brown-staining storage material (arrows) within endoneurial cells, with striking green dichroism seen under polarised light (inset). Ultrastructural examination (E-H) shows hypomyelinated/demyelinated axonal profiles with myelin debris and osmiphilic storage material within the cytoplasm of Schwann cells (E, F). Higher magnification reveals ‘tuff stone’ (G) and ‘prismatic’ (H) inclusions characteristic of sulfatide storage. P27: Immunostaining for CD3 shows few scattered endoneurial cells and occasional endoneurial perivascular clusters (arrow, I). A Toluidine blue resin semi-thin stained section shows moderately oedematous fascicles of uniform appearance (J). Within each fascicle, there is patchy demyelination associated with a prominent hypertrophic Schwann cell response with prominent, frequent onion bulbs (circles, K). P1: Immunostaining for CD3+ shows one of several prominent clusters of endoneurial perivascular inflammatory cells (L). Resin semi-thin sections stained with MBA-BF show massive endoneurial oedema across three fascicles (M). There is considerable axonal loss and the surviving axons show a uniform pattern of hypomyelination surrounded by hypertrophic onion bulbs (N). Scale bar: A, B, I, J, L, M = 100 µm; C, D, K, N = 10 µm

Lumbar puncture was performed in all but 2 cases and majority (*n* = 18/19; 94%) met the CSF criteria [1]. Two had low CSF protein (<0.35 g/l) and one with high CSF cell count (10 white cells). A diagnostic peripheral nerve biopsy (Fig. 2) was performed in 9/21 cases. In 6/9 cases, there was unequivocal evidence of demyelination; of which only three showed a more typical CIDP pattern with patchy demyelination, inflammation and oedema.

Genetic testing for inherited neuropathies was done in half of the cases (*n* = 10) with pre-existing, early or progressive foot deformity and/or poor response to treatment. These included comprehensive 56 gene inherited neuropathy panel (INP) in three and were negative in all cases.

**Fig. 3:**
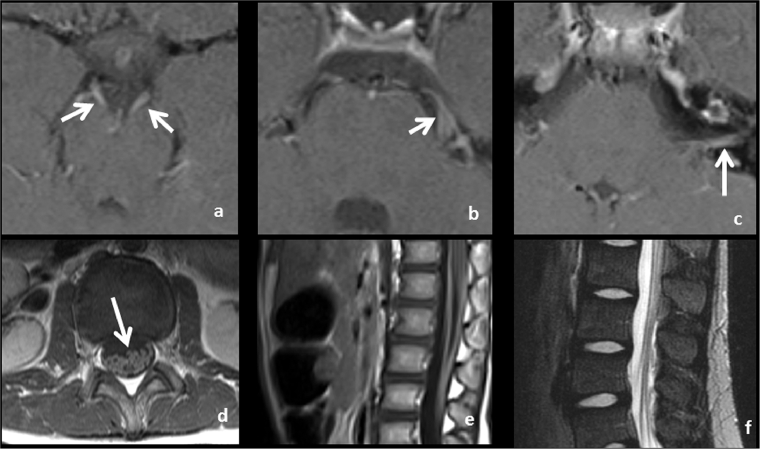
1a–c: Axial contrast enhanced T1-weighted MRI brain sections showing enhancing cranial nerves marked by arrows. 3rd cranial nerves (1a), left 5th cranial nerve (1b) and left 7th and 8th nerves (1c). 1d-f: Axial and sagittal contrast T1-weighted MRI spine sections (d,e respectively) and sagittal T2-weighted MRI (f) shows thickened enhancing cauda equine roots.

Neuroimaging of spine was done in 20 and 9 (45%) had thickening and/or enhancement of cauda equina nerve root (Fig. 3). Two others had incidental thoracic syrinx and degenerative disc changes respectively. Brain imaging was performed in 14 and all were reported as normal except 1 with bilateral 3, 5, 7 and 8 cranial nerve enhancement. Clinically this cranial nerve involvement manifested as facial and bulbar weakness with speech and swallow difficulties and optic neuritis and all these symptoms responded to immunoglobulins and steroids therapy.

**Fig. 4:**
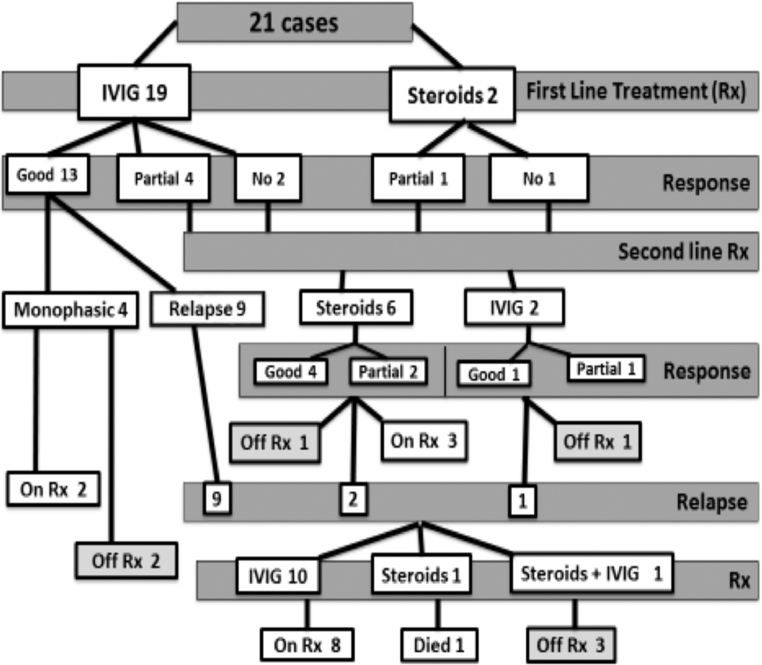
Treatment flowchart.

### 3.3 Treatment (Supplementary table S2, Fig. 4)

All twenty-one children received immuno-modulatory treatment as first line therapy, of which majority (*n* = 19; 90%) received IVIG monotherapy with 68% (*n* = 13/19) showing a good response after first dose. IVIG was then gradually weaned and eventually 6/13 (46%) children with good response to IVIG were off treatment irrespective of disease course (monophasic or relapsing) over a mean FU of 5.5 years (range 1.1–13.7 years). 5 children had side effects including headache, nausea, vomiting and transient high blood pressure but continued on therapy. Two children were started on steroids as first line with partial response in one and no response in other.

Eight children needed second line treatment which was IVIG in two and steroids in six; of these one each in IVIG group had a good or partial response whereas four in the steroid group had a good response to therapy. Those with partial response went on to plasmapheresis in 2 and azathioprine in one case.

43% (*n* = 9) had a monophasic course and 57% (*n* = 12) had a relapsing–remitting course. Of the 12/21 children with relapsing-remitting course, three evolved to chronic progressive. The majority had 2–5 relapses (*n* = 6) and three children each had 1 or more than 5 relapses (*n* = 3) with 3 weeks to 24 months interval between relapses.

IVIG was trialed as first line therapy for relapse in the majority of cases (*n* = 10) and prednisolone was used in the other two. 4 children in this group continued with relapse or progression and were trialed on other immuno-modulatory therapy including plasma exchange (PE), azathioprine, cyclophosphamide and mycophenolate, none of them had a good response except PE in one. Cyclophosphamide was trialed in one child for ten courses with minimal response and no motor worsening. Three children remain on mycophenolate and IVIG infusions with minimal response.

Three children developed adverse effects with steroid use including osteoporosis, triplanar fracture of right ankle and wedge vertebral fracture in one and hypercalciuria and renal calculi in two others; azathioprine caused deranged liver enzymes, lymphopenia and gastrointestinal symptoms in three patients respectively and was stopped.

Of the 21 children, at last follow up, 7 were off treatment, 13 on treatment and 1 child passed away at 14.8 years with H1N1 influenza while on treatment.

### 3.4 Outcome (Supplementary table S2)

At last paediatric follow up 33% (*n* = 7) were off all treatment, of these, two were cured as per CDAS classification (stable and off treatment for more than 5 years) and 5 others were in remission (stable and off treatment for less than 5 years). A total of six (28%) still had unstable active disease and were on treatment.

**Fig. 5:**
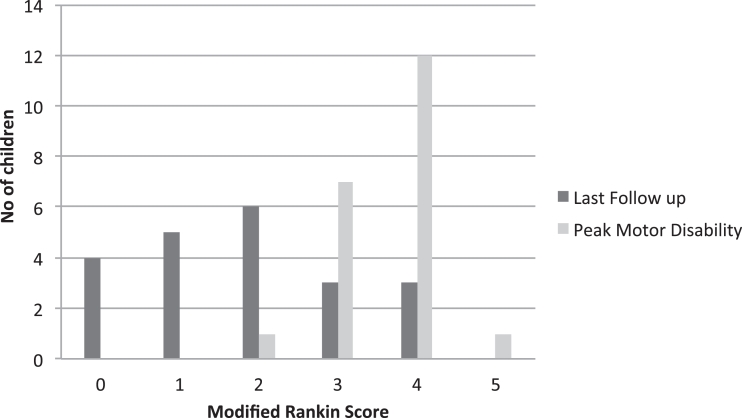
Modified Rankin Score for the group showing more number of children with improved score at last follow up compared to higher number of children with worse scores during peak of illness.

On reviewing the MRS scores (Fig. 5) 13 children had a peak motor disability MRS of 4–5 and this number reduced to 3 children with MRS 4–5 at last follow up (Supplementary Table S2). 43% (*n* = 9) were left with no or minimal residual disability.

### 3.5 Diagnostic revisions difficulties and atypical cases

Supplementary Table S2 highlights the findings in individual cases that led to diagnostic revisions. These included findings like early onset, pre-existing wasting/contractures, associated co-morbidities like development delay, epilepsy, chromosomal deletions, cognitive decline and abnormal investigations like high CK or abnormal metabolic profile, poor or no response to IVIG therapy.

Of the total 30 cases with initial suspicion of CIDP, 19 (Fig. 1) had a re-evaluation of diagnosis either to CIDP or an alternate diagnosis due to new / atypical findings or investigation results or change in disease course. In 10/19 (52.6%) CIDP was the final diagnosis and nine had alternative diagnoses (MLD, MNGIE, mitochondrial, likely genetic neuropathy (*n* = 2) and GBS (*n* = 4). The diagnosis of CIDP is thus very challenging and needs frequent re-evaluation.

Two children in our cohort were later diagnosed with MNGIE and MLD respectively. Both these children met the clinical, CSF and electrophysiological criteria for demyelination and managed as CIDP initially. A routine screen for urine organic acids was abnormal in one and led to a genetic diagnosis of MNGIE two years after presentation.

The other child with MLD presented with unsteady gait and loss of ambulation at 13 months and showed a partial response to IVIG. Nerve biopsy showed presence of granular storage material in cytoplasm of myelinating schwann cell and was crucial in leading to the diagnosis of MLD. Meanwhile, her symptoms progressed with cognitive decline and upper motor neuron signs. Arylsulfatase A activity was very low in white blood cells, and the diagnosis was subsequently genetically confirmed with compound heterozygous mutations in the *ARSA* gene. MRI brain at 2.6 years showed typically tigroid pattern of leukodystrophy with bilateral dark thalami with thickening and enhancement of 3rd, 5th, 7th and 8th cranial nerve and cauda equina nerve roots.

Two siblings born to non-consanguineous parents were thought to have a congenital onset of CIDP. History of polyhydramnios and reduced fetal movements suggested antenatally onset disorder. The index case was born following foetal distress, needing CPAP support for two days. She was profoundly hypotonic, weak with poor antigravity movements and areflexia. At four weeks of age her NCS demonstrated absent sensory nerve action potentials (NAP), motor conductivity (cV) 1–2 m/s and dispersed compound motor action potentials (CMAP). She was treated with 4–5 courses of IVIG following which she had gradual normalization of clinical examination and NCS at 9.5 months of age. Her younger sibling presented similarly with hypotonia and weakness and responded well to IVIG therapy, continuing to make improvement. Her mother when tested did not have a neuropathy.

Two children initially thought to have CIDP were later considered to have a genetic etiology; one among them (Pt 8) presented at 4 years with only lower limb, distal involvement with tip toe gait and thereafter rapidly progressive bilateral equinovarus and calf wasting. Nerve biopsy showed a chronic picture of re and demyelination with no active inflammation, more suggestive of a genetic cause. The other child (Pt 15) had an early presentation and developed a progressive disease course with marked foot deformity. A nerve biopsy was declined. Both the children did not respond to therapy with negative genetic testing including inherited neuropathy panel.

## 4 Discussion

We are presenting data of CIDP cohort from a single tertiary center in UK. McMillan et al have previously reported on 30 American children with CIDP and combined this with data from 11 previous case series (1980–2009) to provide a comprehensive review of 143 childhood CIDP cases [2].

Based on the revised diagnostic criteria for childhood CIDP proposed by Nevo et al. [1], all but 5 cases in our group of 30 met the inclusion criteria. 4 others met the CIDP revised criteria but had alternate diagnosis made and this highlights the difficulty in establishing a CIDP diagnosis despite updated criteria. The updated criteria rely on EDT criteria and 9 of the 21 children did not meet these. While nerve studies can present challenges in children and often less studies might be performed compared to examination of a co-operative adult, in this study at least 4 nerves were examined, if studied at our centre.

The overlap between GBS and acute onset CIDP and between inherited neuropathy and CIDP were the two main common themes in differential diagnosis in our group. Difficulties in diagnosis of GBS and the acute onset CIDP is known (Riekhoff et al. [6]). Children with initial suspicion of GBS (*n* = 9) in our group had diagnostic revision due to relapse, progression or pre-existing wasting or contractures. Due to the chronic nature of symptoms genetic neuropathies can mimic CIDP and can be equally challenging to differentiate the two. Shabo et al. [7] presented retrospective data on 118 children with polyneuropathies to obtain an overview of their etiologies. Hereditary polyneuropathies made up 68%. In our group, hereditary polyneuropathy was raised as a differential diagnosis in 11 but not proven so far.

A clinical suspicion of CIDP along with appropriate tests, treatment, regular follow up and serial electrodiagnostics helped to confirm the diagnosis in our cohort. In cases where there were atypical features either in history, investigations or progression, the diagnosis was revised. This is shown by 52% (*n* = 10/19) of our cohort diagnosed as CIDP on re-evaluation suggesting the challenges in diagnosis of this treatable condition.

The diagnostic pathological differentiation between a genetic versus acquired demyelinating neuropathy is equally challenging, as evidenced from our biopsy series. Three cases had a more confident diagnosis favouring CIDP showed the classic patchy demyelination with three others having a pathological diagnostic ambiguity on account of presence of overlapping pathology between genetic neuropathy versus CIDP or lack of a ‘full house’ e.g. lack of overt inflammation, a more uniform pattern of demyelination and inconspicuous onion bulbs. Biopsies were normal in two cases and showed only acute axonal degeneration in two; of these 2 had a diagnosis of CIDP. This highlights that lack of biopsy evidence does not exclude a demyelinating process.

We had two established metabolic diagnosis (MNGIE and MLD), which were initially diagnosed as CIDP. This has been reported in literature. Bedlack et al. [8] described five patients with genetically confirmed MNGIE neuropathy mimicking CIDP. They were initially diagnosed with CIDP, and three were treated with immunomodulating drugs with poor response. Haberlandt et al. [9] reported on three children with MLD who presented with a demyelinating polyneuropathy in the absence of white matter changes in brain MRI, which can be challenging, as was our case with subtle MRI changes earlier on.

In our study the two siblings thought to have congenital CIDP had a later diagnostic revision to GBS. Congenital presentations of CIDP are rare but have been reported before [10], [11]. Majumdar et al. [11] reported on a neonate with severe congenital CIDP with complete spontaneous resolution and hypothesised an intrauterine neuropathy due to either expression of foetal myelin antigen and/or antibody transfer between mother and foetus, raising a possibility of an immunologically driven injury to the myelin sheath in utero. In our case mother and baby's blood have been sent for further analysis to investigate into antibody-mediated process against the foetal myelin.

Cranial nerve involvement has been reported in children with CIDP and can cause considerable diagnostic confusion. We as clinicians need to be aware of this presenting feature in CIDP. Costello et al. [12] reported on a 17 year old who presented with chronic diplopia, facial weakness and generalized motor weakness and was diagnosed with CIDP. Review article by Riekhoff et al. [6] highlights the eye signs, cranial nerve palsies and bulbar disturbances in children with CIDP and that cranial nerve involvement may be the only presenting symptom.

In our study the treatment response to first line IVIG therapy was deemed as good in 68% of cases; this is comparable to the existing literature with a combined value of 79% from 135 total reported cases by McMillan et al. [2]. IVIG was well tolerated in our group. Steroid therapy was used in fewer cases but relapse of CIDP symptoms after withdrawal of therapy was a major problem. Only a few children were trialed on other immune-modulatory agents and as the numbers were small it is difficult to draw conclusions from this group.

McMillan et al. [2]. highlighted that the combined long term outcome for their cohort and the literature revealed a favourable prognosis for most patients with childhood CIDP. Similar data was also presented by Riekhoff et al. [6]. In our group there was an overall improvement in MRS (Fig. 5) from scores at peak motor disabilty) to scores at last follow up There was no deterioration in MRS score in any child during the follow up period though in 2 children there was no improvement in scores despite treatment. In 5 children there was no disability noted at follow up with complete recovery and this complete recovery in cases of childhood CIDP has been seen in several case reports [6].

## 5 Conclusion

Our review highlights the challenges in the diagnosis and management of paediatric CIDP. In general this is a rare diagnosis but clinical suspicion, early recognition and investigation with prompt management can result in better outcomes of this potentially treatable neuropathy. Our review also highlights that various differential diagnoses must be considered in cases where the disease course changes or new signs evolve.

## Appendix Supplementary materials

Image, application 1
