# Supplementary material for: Clinical spectrum, treatment and outcome of children with suspected diagnosis of chronic inflammatory demyelinating polyradiculoneuropathy
Source: Neuromuscul Disord. 2018 Sep;28(9):757–65. doi: 10.1016/j.nmd.2018.06.001 (PMC6509554; doi:10.1016/j.nmd.2018.06.001)
Supplement: Supplementary file 1 [file mmc1.docx]

**Supplementary Table S1: Clinical presentation and Investigations:**

| **Pt no** | **Sex** | **Preceding illness** | **Onset (A/S/C)** | **Age at onset (yr. mth)** | **First symptom described** | **Sensory symptom**  **Y/N;**  **sym/asym; pos/neg** | **4 limbs/**  **UL/LL; prox/**  **Distal** | **CSF protein (mg/dl) - raised** | **NCS:**  **Evaluation – meets criteria** | **NCS:**  **number of criteria met** | **Nerve Biopsy**  **suggestive of demye-**  **-lination** | **Diagnostic**  **Criteria** |
| --- | --- | --- | --- | --- | --- | --- | --- | --- | --- | --- | --- | --- |
| 1 | M | Y | C | 9.1 | W- weak legs | Y/sym/neg | 4 limb; prox > distal | Y | Y / D | 4 major | Y | **CSF + NCS + biopsy : Confirmed CIDP** |
| 2 | M | Y | C | 8.5 | P- Leg pain, toe walker | Y/sym/pos | LL>UL; prox > distal | Y | Y / D | 3 major |  | **CSF + NCS : Confirmed CIDP** |
| 3 | F | Y | S | 3.3 | B- Ataxia | N | 4 limb; prox > distal | Y | Y / D | 3 major | Y | **CSF + NCS + biopsy : Confirmed CIDP** |
| 4 | F | Y | A | 2.7 | B- walked as if drunk | Y/sym/pos | 4 limb; prox + distal | Y | N / D | 2 major / no supportive |  | CSF : Possible CIDP |
| 6 | M | N | C | 11.6 | W- unsteady due to weakness | Y/sym/neg | LL>UL; prox > distal | Y | Y / D | 3 major |  | **CSF + NCS : Confirmed CIDP** |
| 10 | F | N | C | 11.7 | W- difficulty rising from floor | N | LL>UL; prox > distal | ND | Y / D | 3 major | Y | NCS + biopsy : Possible CIDP |
| 11 | M | N | C | 4 | P- leg pain | N | LL>UL; prox > distal | Y | N / D | 2 major in 1 nerve only |  | CSF : Possible CIDP |
| 12 | M | Y | C | 5.1 | P- painful legs then non-ambulant in 4 wks | N | 4 limb; distal > prox | Y | Y / D+A | 3 major |  | **CSF + NCS : Confirmed CIDP** |
| 13 | F | Y | C | 9.8 | W- reduced power in hips | N | LL>UL; prox > distal | Y | N / D | 2 major / no supportive criteria |  | CSF : Possible CIDP |
| 17 | M | Y | A | 2.5 | W- climbing slopes difficult | N | UL>LL; prox > distal | Y | Y / D+A | 2 major / no supportive criteria | N -After Rx | **CSF + NCS : Confirmed CIDP** |
| 18 | M | N | C | 3.4 | P- pain in calves then non-ambulant in 1 wk | N | LL>UL; distal > prox | Y | N / D | 2 major in 1 nerve only |  | CSF : Possible CIDP |
| 19 | M | N | C | 16 | G- foot drop | Y/asym/pos+neg | LL>UL; distal > prox | Y | N / D | 2 major / no supportive criteria |  | CSF : Possible CIDP |
| 20 | M | Y | C | 5 | W- difficulty climbing stairs, getting off floor | Y/sym/pos | LL>UL; prox + distal | Y | Y / D+A | 3 major |  | **CSF + NCS : Confirmed CIDP** |
| 21 | M | N | C | 13.9 | P- Pain in knee followed by weak toes in 2 weeks | Y/sym/neg | 4 limb; distal > prox | Y | N / A | No criteria met | N- non-contributory | CSF : Possible CIDP |
| 22 | M | N | A | 9.11 | W- legs giving away | Y/sym/pos | 4 limb; prox > distal | Y | Y / D | 3 major |  | **CSF + NCS : Confirmed CIDP** |
| 23 | F | N | C | 2 | W- weak, cannot crawl | N | 4 limb; prox > distal | Y | N / D | 2 major in 1 nerve only | Y | CSF + biopsy : Possible CIDP |
| 25 | F | Y | C | 8.6 | W- lower limb weakness | N | LL>UL; prox > distal | Y | N / D+A | 2 major in 1 nerve only |  | CSF : Possible CIDP |
| 26 | M | N | A | 14.5 | W- weak legs | N | LL>UL; prox + distal | Y | Y / D | 4 major |  | **CSF + NCS : Confirmed CIDP** |
| 27 | F | Y | S | 3.3 | B- decline in balance | N | 4 limb; prox + distal | N | Y / D+A | 4 major | Y | NCS + biopsy : Possible CIDP |
| 29 | F | Y | S | 10.9 | G- tip-toeing, frequent falls, high step gait | N | LL>UL; distal > prox | Y | N / A | No criteria met | Y | CSF + biopsy : Possible CIDP |
| 30 | F | N | A | 11.6 | W- falling, weak | Y/sym/pos+neg | 4 limb; prox > distal | N | Y / D | 3 major | N | NCS : Possible CIDP |
| **Non – CIDP cases** | | | | | | | |  |  |  |  |  |
| 5 | M | N | C | 3 | P-pain in legs – limited walking | N | 4 limb; prox + distal | Y | Y / D |  |  | **CSF + NCS : Confirmed CIDP** |
| 7 | F | N | C | 1 | W- unsteady gait due to weakness | N | 4 limb; prox | Y | Y / D+A |  | N | **CSF + NCS : Confirmed CIDP** |
| 8 | F | N | C | 4.2 | G- tip toe then asymmetric gait with inturning feet | N | LL; distal | Y | Y / D |  | N | Not met clinical criteria |
| 9 | F | N | Cong | 0 | W- profound hypotonia and weakness | N | LL>UL; distal > prox | N | Y / D |  | Failed | NCS : Possible CIDP |
| 14 | F | N | C | 14.11 | P-pain in calves | Y/sym/pos | LL>UL; distal>prox | N | Y / D+A |  | N | NCS : Possible CIDP |
| 15 | M | N | C | 2.5 | W – frequent falls, fatigue | N | LL>UL; distal>prox | NR | Y / D+A |  | refused | NCS : Possible CIDP |
| 16 | M | Y | A | 12.5 | W-weak and wobbly in legs | N | UL>LL; distal>prox | N | Y / D |  |  | NCS : Possible CIDP |
| 24 | F | N | Cong | 0 | W- weak legs | N | LL>UL; distal > prox | ND | Y / D |  |  | NCS : Possible CIDP |
| 28 | F | N | S | 10.3 | G-Awkward gait | N | 4 limb; prox + distal | N | N / D |  | N | Not met any laboratory finding |

Pt. no - Patient number, M - male, F – female, Y – yes, N – no, Onset: A –acute, S – subacute, C – chronic, Cong – congenital, yr – year, mth – month, NCS – nerve conduction study, CSF – cerebrospinal fluid

First symptom: W – weakness, P - pain, B – balance, G – gait difficulty, Sym – symmetrical, Asym – asymmetrical, pos – positive symptoms ; neg – negative symptoms,

Involvement: UL – upper limb, LL – lower limb, Prox – proximal.

ND – not done; NR – not reported; D- demyelinating; D+A – demyelinating and axonal; A - axonal

**Supplementary Table S2: Treatment, course and outcome:**

| **Pt no** | **Treatment trialed** | **Evolution (if CIDP) – 2002** | **Present Rx** | **Worst MRS Peak motor disability** | **Last MRS at FU** | **CDAS classification at last FU** | **Revision of Diagnosis** | **Any new/different findings – leading to revision of diagnosis** | **Main differential** | **Final Diagnosis** |
| --- | --- | --- | --- | --- | --- | --- | --- | --- | --- | --- |
| 1 | IVIG | Relapsing-remitting | monthly IVIG  (Transitioned – off Rx) | 4 | 2 | 5c | Y | Progressive foot deformity impacting gait – surgery + orthosis | CMT / CIDP | **CIDP** |
| 2 | IVIG | Relapsing-remitting | 6 weekly IVIG | 4 | 2 | 3b | Y | High CK – 600-800 | Muscular dystrophy | **CIDP** |
| 3 | IVIG, PE, Pred | Monophasic | Weaning pred | 4 | 2 | 3b | N |  |  | **CIDP** |
| 4 | IVIG | Relapsing-remitting | Off >5 yr | 4 | 0 | 1a | Y | GBS – later relapses | GBS / CIDP | **CIDP** |
| 5 | IVIG | NA | Off Rx | 3 | 1 | NA | Y | Urine organic acids- elevated uracil and thymine.  Plasma, urine purine/pyrimidine analysis ↑ thymidine, deoxyuridine  Thymidine phosphorylase activity undetectable in white blood cells and platelets.  Homozygous mutation in TYMP gene identified | MNGIE | MNGIE |
| 6 | IVIG, Pred | Monophasic | Pred + monthly IVIG | 4 | 3 | 4b | N |  |  | **CIDP** |
| 7 | IVIG, Pred | NA | Off Rx | 4 | 4 | NA | Y | Cognitive decline and upper motor neuron signs  Arylsulfatase A – 1.0 nmol/hr/mg (ref 22-103)  Later MRI brain – leukodystrophy,  Muscle biopsy: granular storage material  Confirmed compound heterozygous mutations in ARSA gene | Neuro-degenerative disorder | MLD |
| 8 | Pred | NA | Off Rx | 2 | 1 | NA | Y | Progressive peroneal muscle wasting and bilateral clawing of toes. Nerve biopsy - chronic picture with no inflammation;  INP negative | CMT/CIDP | Likely genetic |
| 9 | IVIG | NA | Off Rx | 5 | 0 | NA | Y | Responded to IVIG - clinically and NCS | Congenital hypomyelinating neuropathy / CIDP / GBS | GBS |
| 10 | IVIG | Monophasic | Off | 3 | 1 | 2b | N |  |  | **CIDP** |
| 11 | IVIG, Pred | Monophasic | Weaning pred | 3 | 0 | 4a | N |  |  | **CIDP** |
| 12 | IVIG | Monophasic | Off | 4 | 2 | 2b | Y | GBS like presentation (non-ambulant within 4 wks), pre-existing toe clawing – INP negative | GBS / CMT / CIDP | **CIDP** |
| 13 | IVIG | Monophasic | monthly IVIG | 3 | 1 | 3a | N |  |  | **CIDP** |
| 14 | IVIG , Pred | NA | Off | 4 | 3 | NA | Y | Epilepsy, dysphagia, No response to IVIG  Later: CSF lactate high - 2.6;  Muscle biopsy-core-like areas, Respiratory chain enzyme- decreased cytochrome oxidase complex IV activity  POLG, mitochondrial sequencing negative, no evidence of mitochondrial depletion | Multi-system disease / mito-chondrial disorder | Possible mito-chondrial or other multi-system disorder |
| 15 | IVIG, PE | NA | IVIG + PE | 3 | 3 | NA | Y | Epilepsy, foot deformity progressive; No clear cut response with IVIG – now trialled PE; INP negative | CMT / CIDP | Likely Genetic /acquired |
| 16 | IVIG, PE | NA | Off | 4 | 2 | NA | Y | GBS like presentation, Subsequent EMGs showed axonal component with some sensory involvement | GBS / CIDP/ motor axonal neuropathy (acute or chronic) | GBS |
| 17 | IVIG, Pred | Relapsing-remitting | Pred + monthly IVIG | 4 | 2 | 4a | Y | GBS – later relapses | GBS / CIDP | **CIDP** |
| 18 | IVIG, IV MP | Relapsing-remitting | IVIG | 4 | 3 | 5c | Y | GBS like presentation (non-ambulant within 1 wk) – later relapses; younger sibling presented with pain, weakness and paraesthesia, INP negative | GBS / CIDP / CMT | **CIDP** |
| 19 | IVIG | Monophasic | monthly IVIG last – (Transitioned – off Rx) | 2 | 1 | 3b | N |  |  | **CIDP** |
| 20 | IVIG | Relapsing-remitting | 2 monthly IVIG | 4 | 1 | 4b | Y | Progressive foot deformity; INP negative | CMT / CIDP | **CIDP** |
| 21 | IVIG, Pred | Monophasic | Off | 3 | 1 | 2b | Y | Significant foot drop / ankle weakness-transitioned to adults | CMT / CIDP | **CIDP** |
| 22 | IVIG, Pred, Azath | Relapsing-remitting | IVIG +Pred + myco | 4 | 2 | 5c | Y | GBS – later relapses | GBS / CIDP | **CIDP** |
| 23 | Pred, IVIG, Azath, PE, Cyclo, myco | Initially relapsing-remitting then Chronic progressive | pred + myco (Transitioned – on Rx) | 5 | 4 | 5c | Y | early onset, mild motor delay; MRI brain normal, genetic tests for neuropathy negative (no panel) | CMT / CIDP | **CIDP** |
| 24 | IVIG | NA | IVIG 4 weekly | 5 | 2 | NA | N | Elder sibling affected with congenital neuropathy | CIDP/ GBS | GBS |
| 25 | IVIG | Relapsing-remitting | nil-off since >5 yr | 3 | 0 | 1a | N |  |  | **CIDP** |
| 26 | IVIG | Relapsing-remitting | Off | 3 | 0 | 2a | Y | GBS – later relapse | GBS / CIDP | **CIDP** |
| 27 | IVIG, Pred | Initially relapsing-remitting then chronic progressive | Pred weaning dose  (Transitioned – off treatment | 4 | 4 | 5c | Y | chronic musculoskeletal pain and weakness: PMP22 gene mutations, P0 mutation, GJB1 negative (no panel) | CMT / CIDP | **CIDP** |
| 28 | IVIG, Pred | NA | Off | 3 | 1 | NA | Y | GBS - episodes of chronic pain and stiffness | GBS / CIDP | GBS |
| 29 | Pred, IVIG | Monophasic / incomplete | Off | 3 | 3 | 2b | Y | Progressive foot deformity, transitioned to adult – genetic testing negative (no panel) | CIDP / CMT | **CIDP** |
| 30 | IVIG, Pred, Azath, PE, myco | Initially relapsing-remitting then chronic progressive | Pred + myco  (passed away) | 4 | 4 | 5c | Y | GBS – later relapses; Motor delay, chromosomal deletion, seizures,  continued motor deterioration | GBS / CIDP / CMT | **CIDP** |

MRS – modified Rankin scale; FU: Follow up; NA – not applicable (as non-CIDP cases); yr – year; Rx – treatment; Y – yes; N – no; CSF – cerebrospinal fluid, EMG – electromyography, IVIG - immunoglobulins; Pred – prednisolone, PE – plasma exchange, IVMP – Intravenous methylprednisolone, Azath – azathioprine, Cyclo – cyclophosphamide, myco – mycophenolate; GBS – Guillain-Barre syndrome, MNGIE - Mitochondrial Neurogastrointestinal Encephalopathy ; MLD - Metachromatic Leukodystrophy ; CMT – Charcot-Marie-Tooth disease, INP – inherited neuropathy panel
